# Targeting Human Transmission Biology for Malaria Elimination

**DOI:** 10.1371/journal.ppat.1004871

**Published:** 2015-06-18

**Authors:** Sandra K. Nilsson, Lauren M. Childs, Caroline Buckee, Matthias Marti

**Affiliations:** 1 Department of Immunology and Infectious Diseases, Harvard T.H. Chan School of Public Health, Boston, Massachusetts, United States of America; 2 Centre for Communicable Disease Dynamics and Department of Epidemiology, Harvard T.H. Chan School of Public Health, Boston, Massachusetts, United States of America; Institut Pasteur, FRANCE

## Abstract

Malaria remains one of the leading causes of death worldwide, despite decades of public health efforts. The recent commitment by many endemic countries to eliminate malaria marks a shift away from programs aimed at controlling disease burden towards one that emphasizes reducing transmission of the most virulent human malaria parasite, *Plasmodium falciparum*. Gametocytes, the only developmental stage of malaria parasites able to infect mosquitoes, have remained understudied, as they occur in low numbers, do not cause disease, and are difficult to detect in vivo by conventional methods. Here, we review the transmission biology of *P*. *falciparum* gametocytes, featuring important recent discoveries of genes affecting parasite commitment to gametocyte formation, microvesicles enabling parasites to communicate with each other, and the anatomical site where immature gametocytes develop. We propose potential parasite targets for future intervention and highlight remaining knowledge gaps.

## Introduction

Over 200 million people contract malaria each year, with nearly half of the global population at risk of infection [1]. Of the five malaria parasite species that infect humans, *Plasmodium falciparum* is responsible for the majority of the 600,000 malaria-related deaths occurring every year [1]. Despite repeated calls for global malaria eradication in the past century, the local and regional elimination programs that underlie this larger goal have proven to be challenging. In particular, the lack of an effective vaccine and the emergence of drug-resistant parasites suggests that interventions targeting the symptom-causing (asexual) parasite forms are insufficient to interrupt transmission. Indeed, many *P*. *falciparum* infections are not severe, and in highly endemic regions, most cause no symptoms at all but can nevertheless contribute to malaria transmission. The importance of these asymptomatic infections, in particular submicroscopic ones, for onward transmission remains controversial [2], although evidence suggests they contribute significantly [2–5]. With a view to defining the human infectious reservoir, research into the basic biology of parasite transmission, a process that remains poorly understood, has been recently reinvigorated.

**Fig 1:**
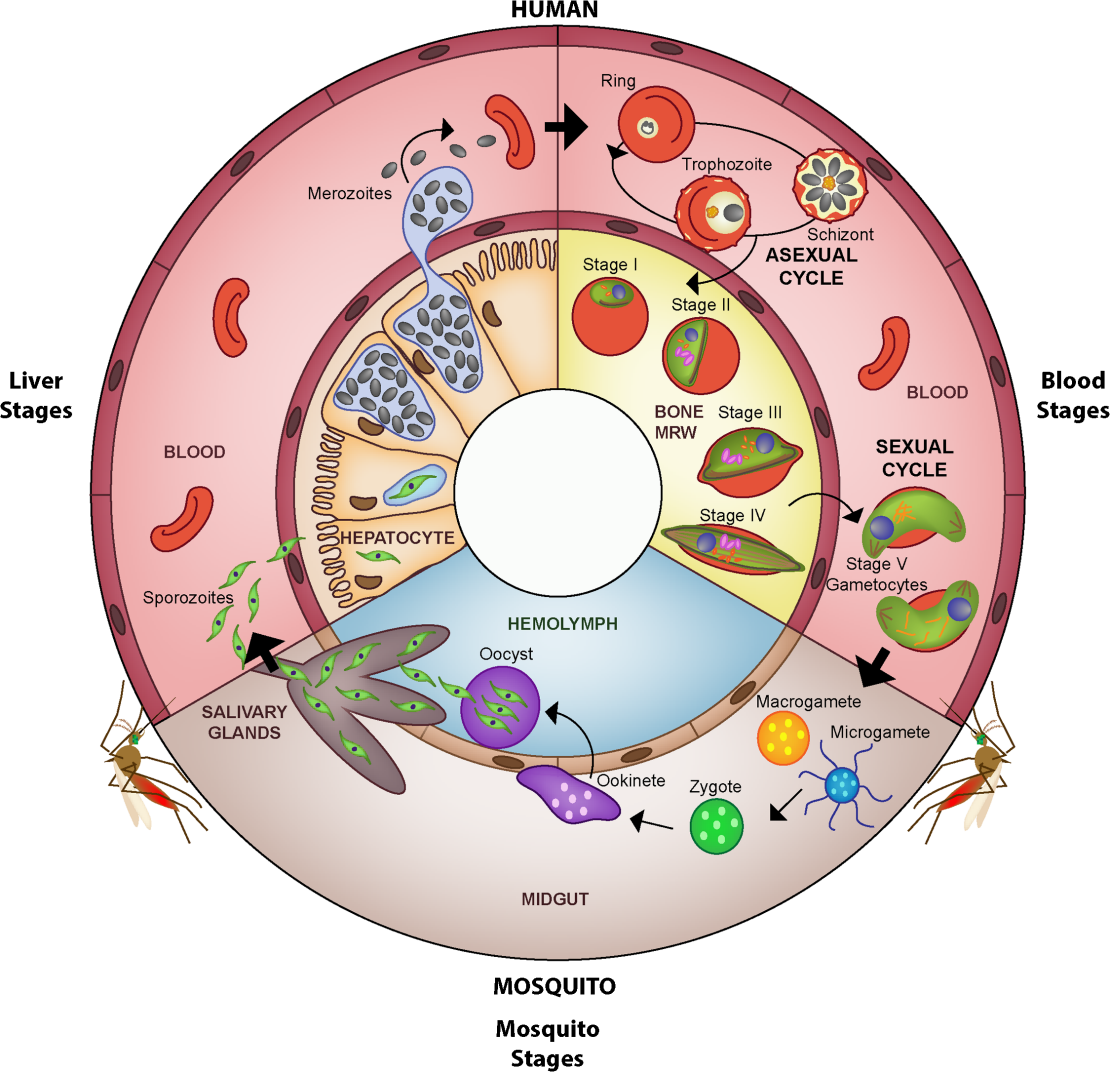
Life cycle of *Plasmodium falciparum*. The malaria parasite is transmitted to the human host when an infected female *Anopheles* mosquito takes a blood meal and simultaneously injects a small number of sporozoites into the skin. After reaching the liver, the sporozoites invade hepatocytes in which they develop into a liver schizont and replicate asexually. After about seven days of liver stage development, each infected hepatocyte releases up to 40,000 merozoites that enter the peripheral blood stream. Once in the blood stream, merozoites quickly invade circulating red blood cells (RBCs), thereby initiating the repeated asexual replication cycle. Over the course of 48 hours, the parasite progresses through the ring and the trophozoite stages before finally replicating into 8–32 daughter merozoites at the schizont stage (schizogony). At this point, the parasitized RBC (pRBC) ruptures and releases merozoites into circulation, commencing another round of asexual replication. Mature asexual stages that display increased stiffness, trophozoites and schizonts, adhere to the vasculature in various organs, which allows them to avoid splenic clearance. During each cycle, a small subset of parasites divert from asexual replication and instead produce sexual progeny that differentiate the following cycle into male and female sexual forms, known as gametocytes. A subset of parasites (see possible scenarios in Fig 4) leave the peripheral circulation and enter the extravascular space of the bone marrow, where gametocytes mature and progress through stages I–V over the course of eight to ten days (gametocytogenesis). Although evidence suggests that the bone marrow is the primary location of gametocyte maturation, some immature gametocytes have been observed elsewhere in the human body, such as in the spleen. By stage V, male and female gametocytes re-enter peripheral circulation, in which they become competent for infection to mosquitoes. Once ingested by a mosquito, male and female gametocytes rapidly mature into gametes (gametogenesis). Within the midgut, the male gametocyte divides into up to eight flagellated microgametes (exflagellation), whereas the female gametocyte develops into a single macrogamete. Fertilization of a macrogamete by a microgamete results in the formation of a zygote, which undergoes meiosis and develops into an invasive ookinete that penetrates the mosquito gut wall. The ookinete forms an oocyst within which the parasite asexually replicates, forming several thousand sporozoites (sporogony). Upon oocyst rupture, these sporozoites migrate to the salivary glands, where they can be transmitted back to the human host during a blood meal. Asexual parasites (in RBCs) are represented in pale yellow, sexual parasites in green.

The life cycle of *P*. *falciparum* is highly complex, involving several developmental stages in both the human host and the mosquito vector (Fig 1). The mature asexual blood stages within the human host are responsible for all the clinical symptoms of malaria, though they are unable to infect the mosquito and complete the parasite’s life cycle. Only the nonreplicating sexual blood stages of the parasite (male and female gametocytes), which circulate at much lower densities and peak at different times during infection than asexual parasites, are capable of developing in the mosquito vector and causing onward infection. Not only are the developmental processes and dynamics of gametocytes in the human poorly understood, making it difficult to correlate asexual parasitemia with gametocyte density [6], but also the association between gametocyte density in the blood and infectivity to mosquitoes appears to be nonlinear [7]. Unlike directly transmitted pathogens, therefore, parasite load per se is a poor indicator of infectiousness, and we currently lack efficient methods to measure the human infectious reservoir that underlies malaria transmission.

In order to target interventions during elimination programs and to design new transmission-blocking vaccines or drugs, we need a deeper understanding of the complex processes that underlie human infectiousness. Here, we will discuss recent advances in *P*. *falciparum* gametocyte biology, describe key knowledge gaps (Box 1) that must be addressed in order to direct future interventions, and identify potential parasite targets that may be explored for development of drugs and vaccines aiming to interrupt malaria transmission. We follow the parasite from commitment along the sexual pathway to hidden development in the human host and finally to transmission to the mosquito vector.

**Box 1. Knowledge Gaps**

**Commitment**

**Fig 2:**
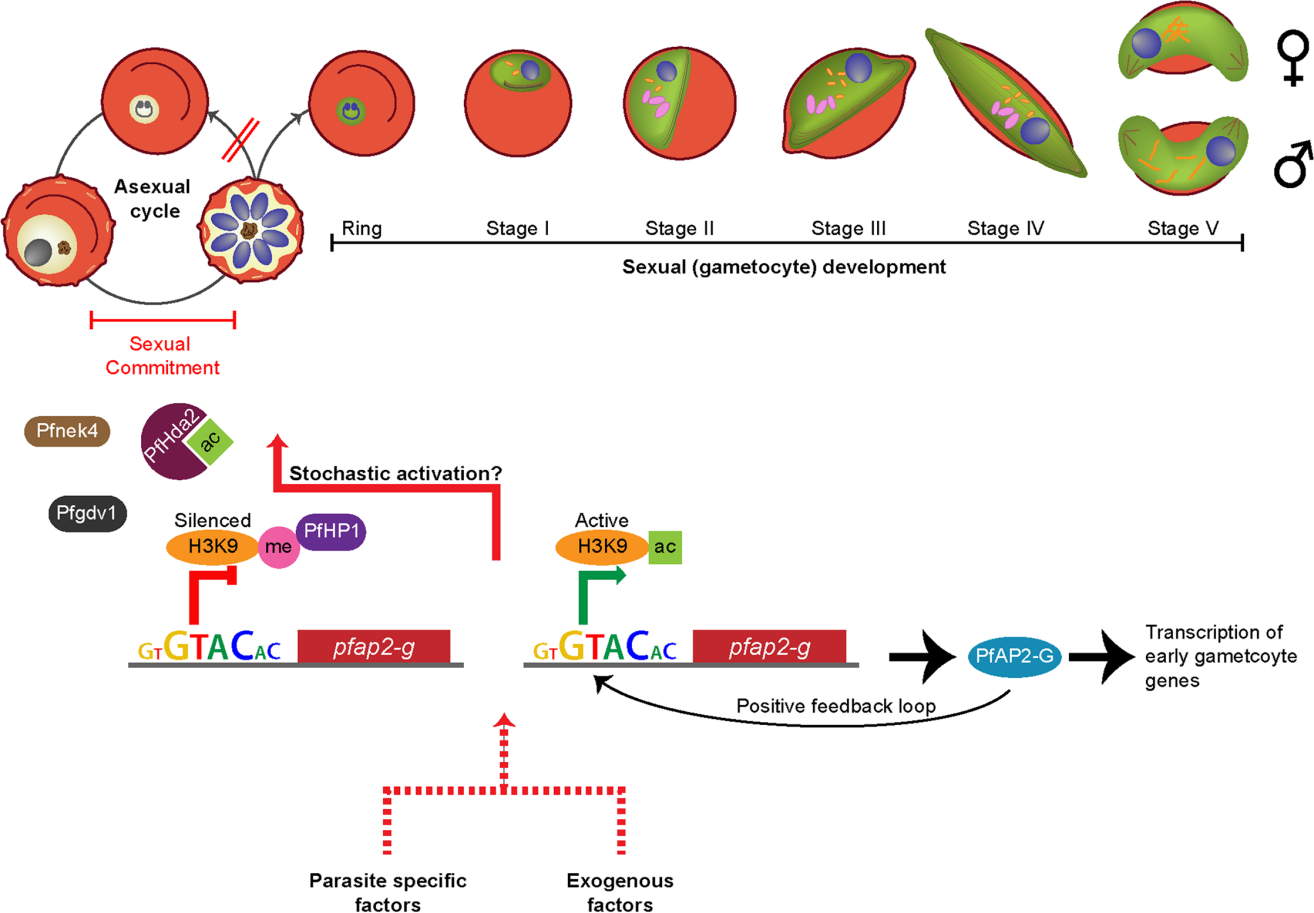
Commitment to sexual maturation. During each asexual cycle within the human blood, a small proportion of parasites cease to replicate asexually and instead produce sexual progeny that develop into nonreplicating gametocytes, capable of onward transmission to the mosquito vector. Commitment to the sexual pathway occurs at a low “baseline” rate during each asexual replication cycle [14,15], and the decision to switch is thought to be made prior to schizogony during the previous asexual replication cycle [10–12]. The switch to gametocytogenesis is governed by the essential transcription factor PfAP2-G (Box 2), which is epigenetically controlled byPfHda2 and PfHP1 [16,17]. PfHda2 likely promotes transcriptional silencing by removing acetyl groups on histones, thereby allowing methylation of histone 3 lysine 9 (H3K9). PfHP1 may subsequently bind H3K9me, leading to heterochromatin formation and *Pfap2-g* repression. The perinuclear protein *Pfgdv1* is another key player that likely operates upstream of PfAP2-G. *Pfgdv1* is expressed in a subset of schizonts, is associated with an increased expression of genes involved in early gametocytogenesis, and has been shown to be critical for gametocyte production [18]. The Nima-related kinase *Pfnek4* may also play a role in sexual commitment, as the kinase is expressed in a subpopulation of schizonts that display a higher sexual conversion rate [19]. *Pfnek4* is, however, not strictly gametocyte specific and can be genetically deleted without affecting gametocyte formation [19]. Although constitutively silenced in asexual parasites, *Pfap2-g* could be prone to stochastic activation, resulting in a low level of gametocyte formation. Once initiated, the transcription of *Pfap2-g* may be further activated via a positive feedback loop. The baseline conversion rate can be altered by various factors: those that are derived from the parasites themselves (found within conditioned medium from high-parasitemia in vitro cultures) and those that are exogenous (antimalarial drugs, anemia, elevated reticulocyte levels, and host immune factors). Enhanced conversion by external factors is extensively reviewed elsewhere [27]. It has recently been demonstrated that the conditioned medium effect is the result of cellular communication within the parasite population via pRBC-derived microvesicles (Box 3) [24,25].

- What factors activate the Apicomplexan Apetala 2 (ApiAP2)-mediated sexual conversion pathway (Fig 2)?
- How are stress and the microvesicle pathway linked to epigenetic switch rates (Section 2)?
- Which components within microvesicles modulate sexual conversion (Box 3)?
- What drives reproductive restraint in *P*. *falciparum*, other *Plasmodia*, and *Hemosporidia* (Section 2)?
- What determines the sex ratio, and how is it modulated (Section 2)?

**Sequestration**

- How do gametocytes come to be in the bone marrow (Section 3, Fig 4)?
- Do sexually committed merozoites preferentially invade young RBCs, whether reticulocytes in the vasculature or RBC progenitor cells in the extravascular compartment of the bone marrow (Fig 4)?
- Does the presence of parasites and/or gametocytes in the bone marrow induce pathology of the hematopoietic system, such as dyserythropoiesis (Section 3)?
- Is the bone marrow associated with immune tolerance, preventing immune recognition of parasites (Section 3)?
- How does the mature (stage V) gametocyte exit the bone marrow and return to circulation? Can the parasite modulate the timing of exit (Section 3, Fig 4)?

**Transmission**

- What is the best way to detect and measure infectious gametocytes (Section 4)?
- What is the contribution of subdermal localization and aggregation of mature gametocytes towards infection (Section 4)?
- How can gametocyte load be used to predict infectiousness to feeding mosquitoes (Section 4)?
- How do the outcomes of feeding assays relate to human infectious reservoir (Section 4)?
- What immune responses exist towards different stages of gametocytes in the human (Section 4)?

## Developmental Decisions: To Proliferate or to Transmit?

The first step in the transmission of malaria parasites from the human host to the mosquito vector is the formation of male and female gametocytes. At this juncture, the parasite exhibits adaptive strategies with respect to two aspects of gametocyte formation: (i) the proportion of asexual parasites that develop into gametocytes, referred to as the conversion rate [8] and (ii) the ratio of male to female gametocytes formed [9]. The precise point within an individual parasite’s life cycle at which it commits to the sexual pathway and determines the ensuing gametocyte sex ratio remains unclear but is thought to occur prior to merozoite formation [10–13]. The molecular mechanism responsible for the switch from asexual to sexual development has remained elusive until the recent identification of a master gene, *ap2-g*, responsible for triggering a transcriptional cascade that initiates gametocytogenesis in both *P*. *falciparum* [14] and *P*. *berghei* [15], a malaria parasite of rodents (Box 2). This conserved member of the AP2-family of transcription factors appears to be generally epigenetically silenced in asexual parasites by *P*. *falciparum* histone deacetylase 2 (PfHda2) and *P*. *falciparum* heterochromatin protein 1 (PfHP1) but could be prone to stochastic activation, leading to the low level of “background” gametocyte formation commonly observed in vitro (Fig 2) [16,17]. Potential upstream mediators of the AP2 master switch include the schizont expressed proteins *P*. *falciparum* gametocyte development 1 (Pfgdv1) and Nima-related kinase (Pfnek4), of which the former has proven to be crucial for gametocyte formation [18,19]. At a population level, the timing of gametocyte appearance in asymptomatic and symptomatic infections is poorly understood.

**Box 2. Epigenetic Control of Gametocyte Formation**

Prior to the discovery of the *Apicomplexan* AP2 (ApiAP2) family of DNA-binding proteins, it was thought that *Plasmodium* parasites had a minimal set of transcription factors [101]. ApiAP2 proteins have been found to regulate a variety of developmental processes along the parasite cycle, including ookinete formation [102], sporozoite maturation [103], and development within hepatocytes [104]. AP2-G, a conserved member of the ApiAP2 transcription factor family, was recently identified as an essential regulator controlling the switch from asexual to sexual development in both *P*. *falciparum* [14] and *P*. *berghei* [15]. In *P*. *falciparum*, the *Pfap2-g* locus appears to be in an epigenetically silenced state in asexual parasites but could be prone to stochastic activation explaining the commonly observed low level of gametocyte formation under standard, noninducing culture conditions (Fig 2) [14]. Interestingly, recent evidence suggests that the expression of *Pfap2-g* could be mutually exclusive with that of *var* genes, the gene family encoding the major asexual surface ligand PfEMP1 responsible for asexual sequestration [14]. Indeed, PfHda2, an important histone-modifying enzyme involved in *P*. *falciparum* gene silencing, modulates not only expression of multigene families such as *var*, *stevor*, and *rifs*, but also sexual commitment by promoting transcriptional silencing of *Pfap2-g* [17]. Similarly, PfHP1, a regulator of heritable gene silencing, controls the mutually exclusive expression of antigenically varying genes and also regulates sexual conversion by maintaining *Pfap2-g* in an epigenetically silent state [16]. Taken together, these novel findings indicate that the decision between continuing to proliferate asexually and differentiating into nondividing but transmissible gametocytes is under transcriptional and epigenetic control and suggest a link between transmission and virulence.

Beyond this baseline rate of gametocyte formation, environmental modulation or “stress” (a poorly defined term used to indicate a variety of external factors detrimental to the parasite) has been reported to cause additional asexual parasites to commit to the sexual pathway [20,21]. A commonly used method to produce gametocytes has been developed based on this hypothesis [22] but has not been formally tested for an effect on conversion rate. It was thought that some unidentified parasite-derived factor found in this conditioned medium provides a stress signal that causes a subset of parasites to withdraw from the recurrent asexual replication and instead form gametocytes [23]. Microvesicles, derived from the membrane of parasitized red blood cells (pRBCs), have recently been shown to be the critical factor in conditioned media capable of inducing increased conversion to gametocytes [24,25]. Other putative triggers such as elevated levels of immature RBCs, serum, and immune cells from naturally infected donors have yet to be confirmed (Box 3, Fig 2) [6,26–29]. While the precise pathway of the microvesicle-mediated initiation of gametocytogenesis remains unresolved, it may occur via AP2-G or another member of this family of transcription factors. Microvesicles seem to be a method by which parasites communicate, initiating a response to high parasitemia or other unfavorable conditions and enhancing the prospect of rapid transmission to a new host [24,25]. A sensing mechanism of this type is likely to be of particular importance for pathogens that cause long, chronic infections, as they need to adapt to fluctuations in the host environment. Indeed, another protozoan parasite causing chronic infections, *Trypanosoma brucei*, has a density sensing mechanism associated with a life cycle switch [30–32].

**Box 3. Communication between Parasites**

Despite the ubiquitous in vitro usage of parasite-conditioned medium to induce gametocyte formation, it was unknown until recently that microscopic vesicles in conditioned media represent a critical factor responsible for the observed increase in gametocyte formation [24,25]. Microvesicles are released by both uninfected and parasitized RBCs, although the latter release about ten times more per cell and are the only ones capable of uptake of microvesicles [24]. Interestingly, recent work demonstrated that pRBC-derived microvesicles promote sexual differentiation, arguing that parasites are able to sense their environment and respond accordingly [24,25]. The identity of the factor(s) in microvesicles responsible for stimulating the increased sexual commitment remains elusive, but it must either directly or indirectly affect the transcriptional program of the parasite, potentially through the Pfgdv1/PfHda2/PfHP1/ApiAP2 pathway (Box 2 and Fig 2). Indeed, internalized microvesicles were observed in close association with the parasite nucleus [24], and transfer of plasmid DNA into the parasite nucleus has been shown [25]. The ability of the parasites to communicate is likely of great importance for the parasite to optimize the balance between transmission success and persistence.

*P*. *falciparum* is unique among *Plasmodium* species in its low epigenetic conversion rate in vitro [8,33] and extended gametocyte maturation time [34,35]. While conversion rates cannot be directly quantified during natural infections, the gametocyte density in experimental *P*. *falciparum* infections is often very low [6,36,37], consistent with conversion rates of 1% to 5% of pRBCs in quantitative models [38,39] and low levels during in vitro experiments under noninducing culture conditions [8,40]. Several plausible hypotheses have been proposed for “reproductive restraint,” the observed low gametocyte density in vivo [41], such as minimizing the immune response directed specifically towards gametocytes or reducing the parasite burden in mosquitoes [41,42]. These hypotheses are consistent with the observed low gametocyte density in vivo, although neither are specific to low conversion rates, since low gametocyte densities can result from both very low and very high conversion rates [41]. In the latter case with high conversion rates, the asexual parasite population is not able to grow to high density, limiting the absolute level of gametocytes possible. Alternatively, competition between parasite genotypes within the human host may generate a low conversion rate in *P*. *falciparum*, if survival through asexual growth is prioritized over gametocyte production [41]. Although studies with the rodent malaria parasite *P*. *chabaudi*, with its shorter gametocyte maturation period, show evidence of a reduced gametocyte conversion rate in the presence of multiple genotypes [43–45], there is no evidence of reduced conversion between genotypes of human *Plasmodium* species. Mixed infections, however, between *P*. *falciparum* and *P*. *malariae* show evidence of increased gametocyte production [46], consistent with enhanced conversion to gametocytes during stress.

The gametocyte sex ratio is another important developmental decision that varies among *P*. *falciparum* parasites [47,48] and could provide a target for transmission-reducing interventions. Parasites ensure that fertilization will succeed within the mosquito through production of a sufficient number of male microgametes to fertilize all the female macrogametes. The ratio of their precursors, male and female gametocytes, has been well studied theoretically for *Apicomplexan* parasites [45,49,50], and in particular for *Plasmodium* [45,51,52]. Evolutionary theory suggests that sexual differentiation will be biased towards production of female gametocytes to ensure an equal ratio of micro- and macrogametes (since male gametocytes form up to eight microgametes upon activation in the mosquito midgut, Fig 1) [53]. Intriguingly, evolutionary theory suggests that the parasites would benefit from modulation of their sex ratio, increasing the proportion of male gametocytes when there is greater opportunity for outbreeding during mixed infections in the mosquito midgut [54]. Indeed, elevated fractions of male *P*. *falciparum* gametocytes have been observed in infections comprised of multiple genotypes as compared to single genotype infections [55]. Although it has been suggested that merozoites from a single schizont are destined to form either all male or all female gametocytes [12,13], the same studies actually observed mixed progeny emerging from the same schizont [10–12]. The developmental mechanisms underlying the shift in population-level gametocyte sex ratios are only beginning to be elucidated, with recent work demonstrating the requirement of Pfmdv1 [56] and PfPuf2 [57] for male gametocyte development. With improvements in detection methods such as single-cell analyses, it will be possible to revisit this and other important developmental questions.

Recent breakthroughs in our understanding of the molecular mechanisms responsible for commitment to the sexual pathway offer new insights into ways to obstruct or prevent infectiousness to mosquitoes [14,15,18,24,25]. The identification of a transcriptionally regulated mechanism responsible for sexual commitment provides an opportunity to reduce or even abrogate the stochastic activation of the sexual pathway by blocking these key molecular players (Fig 2). Moreover, the discovery of how parasites communicate (Box 3), conceivably about conditions that are unfavorable for asexual growth, has opened up opportunities to specifically target sexual commitment by preventing parasite communication. For example, it may be possible to target parasite-derived microvesicles for vaccines, thereby blocking their ability to enhance sexual conversion rates.

## Gametocyte Sequestration: Where Do They Hide?

**Fig 3:**
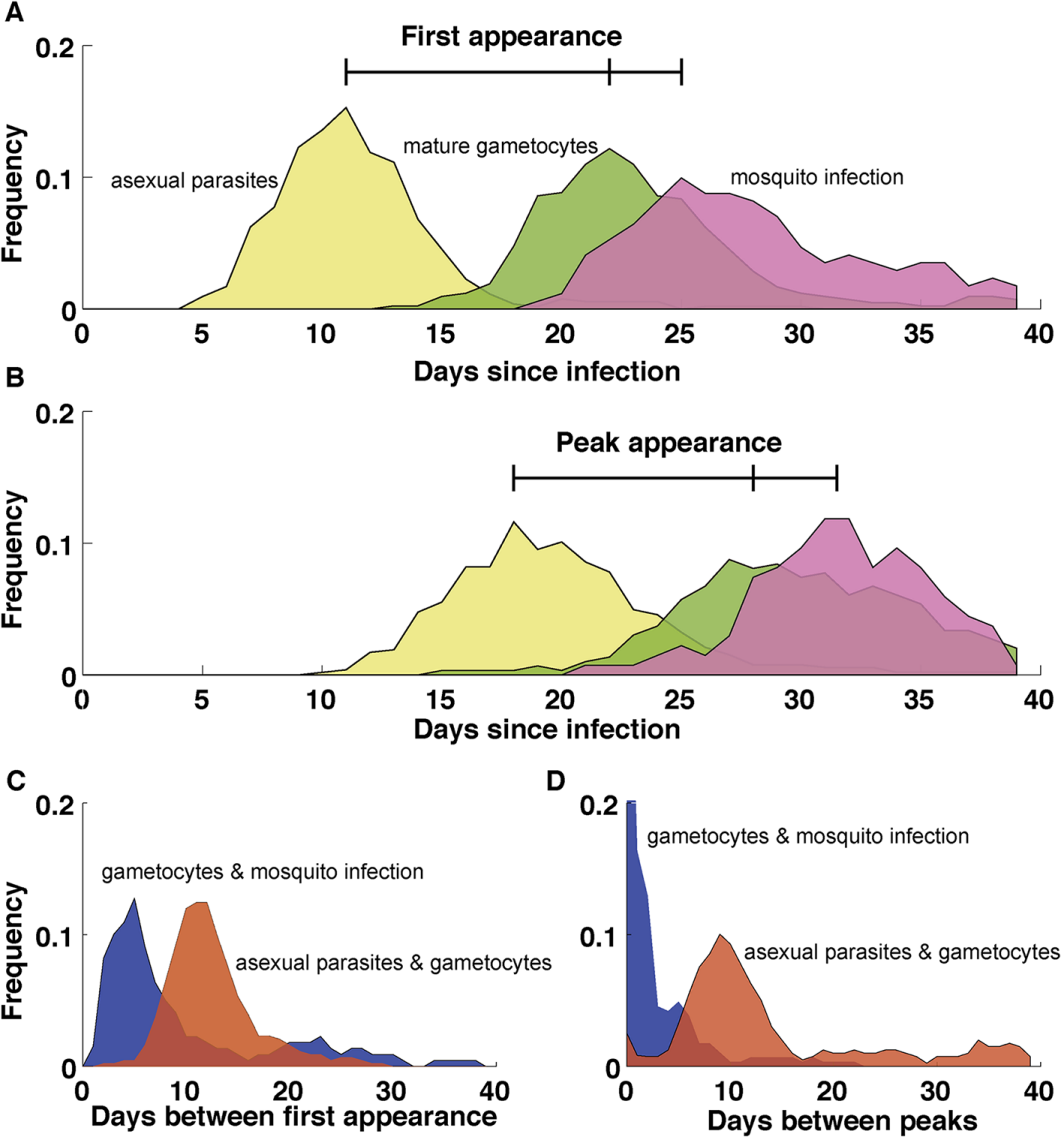
Dynamics of *P*. *falciparum* in malaria therapy patients. (A) The first appearance of patent parasitemia (pale yellow) generally occurs at day 11 following an infectious bite, while gametocytemia (green) arises 11 days later because of the extended development and sequestration of *P*. *falciparum* gametocytes. Mosquitoes (pink) first become infected three days after the appearance of gametocytes. (B) The separation of timing of peaks in parasitemia (pale yellow), gametocytemia (green), and mosquito infection (pink) are similar to their first appearance but delayed by a week. (C) The period between the first appearance of patent parasitemia and gametocytemia (red) centers around 11 days, with some infections requiring as many as 20 days before patent gametocytemia after parasitemia has been observed. Mosquito infection is first observed in the initial days following patent gametocytemia (blue) but may not happen until weeks later. (D) The period between the peak of asexual parasitemia and gametocytemia (red) follows a similar appearance to the timing between initial appearances in C, but with more infections showing weeks between the peak in parasitemia and gametocytemia. The peak in mosquito infection (blue), however, occurs shortly after the peak in gametocytemia. *n* = 106. Patients without gametocytes or mosquito infection do not contribute to the time of appearance or peak of gametocytes and mosquito infection, respectively. Data in A and B are smoothed using a moving average including 5 consecutive days. (Data are courtesy of Dr. William E. Collins and Dr. Geoffrey M. Jeffery.)

Gametocytes are difficult to quantify in vivo since they sequester in tissues during the majority of their maturation [58–60]. Some of the most detailed in vivo dynamical data come from the early 20th century when malaria was used as a therapy for tertiary neurosyphilis [36–39]. Parasite and gametocyte densities were recorded daily for each patient with some infections lasting for hundreds of days [36]. In these infections, the peak density of mature gametocytes occurred around day 40 of patent parasitemia, approximately 13 days after the peak in asexual parasitemia (Fig 3), consistent with in vitro experiments in which gametocytes mature in eight to ten days and with previous theoretical work [61]. In some infections, gametocytes appeared as early as 13 days post–sporozoite inoculation, suggesting that gametocytes can form immediately upon release of merozoites from the liver into the blood stream (Fig 3).

Several studies have observed immature gametocytes in the human bone marrow [59,60,62], but not in highly vascularized organs such as the heart, brain, and placenta [63–66], a strikingly different pattern from the sequestration of asexual stages. A recent quantitative analysis further confirmed the enrichment of immature gametocytes in bone marrow [67], although a secondary reservoir in the spleen could not be excluded. In addition to the distinct anatomical sequestration sites of asexual and gametocyte parasites, asexual pRBCs adhere to vascular endothelial cells but remain in the vasculature while some of the immature gametocytes develop in the extravascular space of the human bone marrow, a site where hematopoietic stem cells mature into RBCs [67,68]. Within this space, most gametocytes were found in close association with erythroblastic islands, structures that facilitate the development of cells of the erythroid lineage [69]. Interestingly, conditions such as anemia and dyserythropoiesis are associated with elevated gametocyte levels, demonstrating a potential interaction between gametocytes and the production of healthy RBCs within the hematopoietic system [70]. Despite the observed enrichment of gametocytes in the human bone marrow, successful in vitro culture of gametocytes demonstrates that their maturation does not require conditions unique to the bone marrow and is possible in both mature and immature RBCs.

There are potential benefits for extravascular sequestration of gametocytes: presence of a nutrient-rich environment with an abundance of young RBCs and potential reprieve from human immune responses. As gametocytes appear to thrive in blood rich with young RBCs [26,71–74], maturation within this main site for RBC production provides the parasites with an ample source of young host cells to invade. Although increased levels of young RBCs are associated with enhanced gametocyte densities, it remains unclear whether sexually committed merozoites preferentially invade RBC progenitor cells [28,67]. During development in the bone marrow, *P*. *falciparum* gametocytes may procure protection from the immune system. Recent in vivo data indicate that gametocytes are significantly less susceptible than asexual parasites to phagocytosis in the bone marrow [67,68], confirming previous in vitro data [75]. Similarly, a recent report indicated that latent *Mycobacterium tuberculosis* resides in the human bone marrow mesenchymal cells and hypothesizes that the bone marrow functions as a protected niche for the nonreplicating form of the bacteria [76].

**Fig 4:**
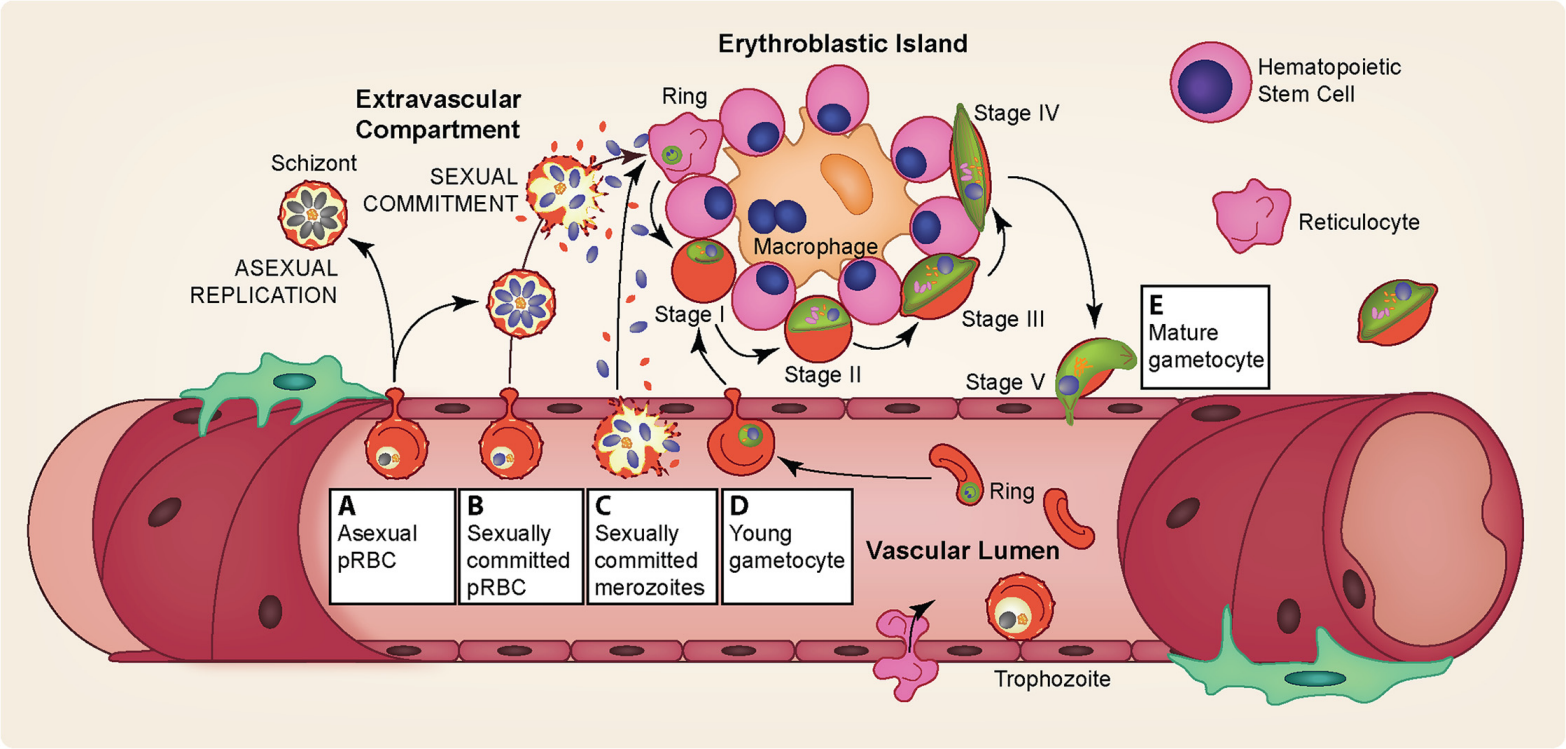
Gametocyte sequestration in the bone marrow. Several pathways may explain the enrichment of gametocytes in the extravascular compartment of the bone marrow and their subsequent release. (A) Asexual pRBCs adhere to the bone marrow endothelium and transmigrate into the bone marrow extravascular space. Once inside the bone marrow, asexual parasites either continue maturation producing asexual progeny or commit to production of gametocytes in the next cycle. (B) Sexually committed pRBCs specifically “home” to the bone marrow sinusoids by adhering to the bone marrow endothelium, after which they transmigrate into the extravascular space. Once in the extravascular compartment, the sexually committed pRBCs undergo schizogony resulting in the release of gametocyte-fated merozoites, which invade the abundant erythroid progenitor cells and for the most part develop attached to erythroblastic islands. (C) Similarly, sexually committed pRBCs could home to the bone marrow but not transmigrate into the extravascular space, perhaps because of their adhesion to the bone marrow endothelial cells or their low deformability. Upon schizont rupture within the bone marrow vasculature, the gametocyte-fated merozoites could enter the extravascular compartment and invade the erythroid progenitor cells. Merozoites from noncommitted asexual pRBCs may also enter the bone marrow and invade erythroid progenitor cells, either continuing asexual replication or forming gametocyte-fated merozoites the following cycle (not pictured). (D) Sexually committed pRBCs may not display a binding preference for bone marrow endothelial cells. Instead, sexually committed pRBCs may be formed in various asexual sequestration sites throughout the body with subsequent invasion of gametocyte-fated merozoites occurring in circulation, in a similar fashion to asexual invasion. Following intravascular invasion, however, the young gametocytes home to the bone marrow sinusoids and adhere to the endothelial cells, after which they transmigrate into the extravascular space. (E) Immature gametocytes display a markedly increased cellular rigidity [79–81]. Upon maturation, however, deformability is rapidly restored, likely allowing the mature stage V gametocytes to exit the extravascular compartment and return to circulation, where they can be taken up by a feeding mosquito [79–81].

It is currently unclear how immature gametocytes reach the bone marrow: whether gametocyte commitment and invasion occurs in the extravascular environment (causing gametocyte progeny to be born there) or young gametocytes home to the bone marrow and subsequently enter the extravascular space (Fig 4). As it has been demonstrated that gametocytes cannot cytoadhere efficiently to endothelial cells [77,78], a switch in cellular rigidity during both the initial and final stages of gametocyte maturation provides a potential explanation for how immature gametocytes are able to enter and mature ones are able to exit the bone marrow extravascular space [79–81]. Indeed rigidity-mediated gametocyte retention in the extravascular space could serve as a potential mechanism for the extended gametocyte maturation of *P*. *falciparum*.

The sequestration of immature gametocytes from peripheral circulation during their prolonged development [58–60] provides potential targets for interventions at multiple points: the entrance into the extravascular space, the maturation within the bone marrow, and the re-entry into the peripheral circulation. For example, targeting surface antigens on pRBCs homing to the bone marrow could prevent parasites from even reaching this site. During development within the bone marrow, the close association of immature gametocytes with erythroblast islands (Fig 4) may play an important role in their maturation [67], and blocking this interaction by targeting potential gametocyte surface antigens could hinder proper gametocyte development. Drugs that prevent the rigidity switch during the end of maturation could potentially trap the gametocytes. Elucidation of the mechanistic basis and molecular factors involved in bone marrow homing, sequestration, and release will likely lead to ways of preventing successful gametocyte maturation and thereby reduce the reservoir of transmissible stages.

## Determinants of Infectiousness to the Mosquito Vector

The nonlinear association between different parasite life cycle stages [6]—from asexual parasites in the human blood to sporozoites in the salivary glands of the mosquito—makes it difficult to use molecular data to determine the human infectious reservoir. Upon release from the extravascular compartment of the bone marrow, mature gametocytes appear to remain in circulation for around six days [38,82] and may require several days in circulation before becoming infectious to the mosquito (Fig 3) [36,83]. While in circulation, mature gametocytes must pass through microvasculature in the dermis in order to be accessible to feeding mosquitoes [84], but it remains to be seen whether mature gametocytes aggregate or preferentially localize in subdermal tissues to increase the likelihood of transmission [85]. Once ingested in a blood meal, both male and female gametocytes must be present for fertilization to occur. While the probability of transmission to the mosquito vector generally increases with higher gametocyte densities [7,86,87], mosquito infection has been observed at submicroscopic gametocyte levels [2–4,88]. These observations suggest that transmission is highly efficient even at very low gametocyte densities and that quantifying mature gametocytes capable of infecting mosquitoes remains challenging despite recent advancements in gametocyte detection [89].

Infectiousness of a *P*. *falciparum–*infected individual to a mosquito vector is difficult to reliably quantify by molecular measurements because of the low number of gametocytes detectable in peripheral blood [6,34,88] and the nonlinear relationship between mature gametocyte densities and oocyst presence in the mosquito [7]. Several different types of feeding assays are currently employed to measure infectiousness, but only direct skin feeding accurately recapitulates the environment during a normal blood feed [5]. For ethical reasons, this method is restricted to older children and adults, excluding the group with the highest prevalence of gametocytes [6,90,91] and thus limiting the scope of direct feeding as a tool for quantifying the infectious reservoir on a population level. The direct membrane-feeding assay (DMFA), in which mosquitoes feed on blood from a naturally infected gametocyte carrier through a membrane, is most commonly used, despite the unnatural transmission method [5]. The standard membrane-feeding assay (SMFA), in which mosquitoes are fed on gametocyte-rich cultured blood through a membrane and dissected to quantify oocyst burden following a gestation period, is the standard to test transmission-blocking activity in the lab [5,88,92]. SMFA, however, is an unnatural transmission method and often underestimates the infectivity of gametocytes [5]. Furthermore, all methods assume that the presence and level of oocysts in the mosquito midgut is indicative of mosquito infectivity. This relationship was recently demonstrated to be consistent for low-density infections despite a portion of the oocysts failing to release viable sporozoites [93,94]. Even in the cases in which oocysts burst and sporozoites successfully migrate to the salivary glands, not all bites from an infected mosquito are equally infectious. In general the heterogeneity in parasite maturation in the mosquito and the lack of standardized protocols for feeding assays makes it difficult to predict transmission efficiency from gametocyte levels [5,7].

Quantifying infectivity is also hindered by both human and mosquito immune responses, as well as competition the parasite encounters within the mosquito vector. The immune system of the mosquito itself must play a role in elimination of the malaria parasite, although the molecular determinants of mosquito protection are poorly understood [95,96]. Transmission-blocking immunity, in which gamete-specific antibodies present in the circulating blood of the human host are taken up in the blood meal and alter the development of parasites within the mosquito, potentially contributes to the lack of correlation between gametocytes and transmissibility [5,97]. Competitive interactions between different pathogens within the mosquito could also be potential factors affecting parasite development. Initial experiments in which *Anopheles* mosquitoes infected with the maternally inherited symbiotic bacteria *Wolbachia* fed on gametocyte cultures have demonstrated that *Wolbachia* significantly inhibits parasite development, although by an unknown mechanism [98].

The portion of the parasite life cycle in the mosquito and the bottlenecks experienced during the transfer of parasites from the human host to the mosquito vector and back have been touted as the most effective intervention targets for over a century. In addition to conventional methods such as insecticides to kill mosquitoes and gametocytocidal or sporonticidal drugs to remove the parasites in the human and vector hosts, novel strategies of intervention are needed. A recent finding that treating mosquitoes with a naturally occurring mosquito hormone that prevents them from mating may be an alternative way to control mosquito populations as insecticide resistance spreads [99]. Within individual mosquitoes, compounds, such as kinase inhibitors or antimicrobial peptides that enhance the mosquito’s ability to fight the parasite, could reduce the prevalence of parasites within mosquitoes. Competition within the mosquito is also a promising intervention, following the recent discovery of *Anopheles* populations naturally infected with *Wolbachia* [100] and the reduction in malaria parasite development observed in experimentally infected *Anopheles* populations [98].

## Conclusion

Reductions in malaria-related morbidity and mortality over the past few decades have shifted the focus of research from attenuation of disease towards strategies to reduce transmission as part of elimination and eradication campaigns. Without a highly effective vaccine, combinations of malaria prevention and treatment are required, and the sexual stage of the parasite’s life cycle represents a key bottleneck and an ideal target for novel strategies. Recent developments in the basic biology of *P*. *falciparum* transmission, including the identification of genes affecting parasite commitment to gametocyte production, microvesicles enabling the parasites to communicate among themselves, and the anatomical site where immature gametocytes develop, have led to the identification of novel intervention targets in addition to more established transmission-blocking strategies. We highlight key knowledge gaps that need to be addressed for the successful development of drugs and vaccines that interrupt malaria transmission. More fundamental research on gametocyte biology will enhance not only our understanding of the basic principles underlying parasite transmission but also our ability to identify infectious individuals.
